# An Updated Classification of Hydraulic Cements Used in Dentistry

**DOI:** 10.1111/iej.70180

**Published:** 2026-05-10

**Authors:** Josette Camilleri

**Affiliations:** ^1^ Dentistry, College of Medicine and Health University of Birmingham Birmingham UK

**Keywords:** additives, clinical interactions, hydration, hydraulic cements, material classification

## Abstract

Mineral trioxide aggregate (MTA) was the first hydraulic cement used in endodontics. It is composed of Portland cement, a binder in concrete, and bismuth oxide to enhance the radiopacity. MTA was introduced in dentistry to be used as a root‐end filling material and also to repair root perforations. The specific use was motivated by the well documented hydraulic nature of Portland cement used in construction. Currently, dental hydraulic cements are used for all endodontic procedures. They are composed of a hydraulic cement with various additives and the material chemistry changes when placed in different clinical fields. These characteristics influence the clinical performance of the materials. A classification of hydraulic cements used in dentistry has already been published, but it is unfortunately already outdated due to the rapid changes in the market and material availability. The aim of this publication is to guide the users of hydraulic cements in dentistry in choosing the right material for the clinical technique being undertaken. The new classification being proposed subdivides the components into the cement which is the binder, the radiopacifier, vehicle, and additives. Irrespective of the cement chemistry, the cements are either calcium hydroxide releasing or not, the vehicle is water or alternative, and the additives are cementitious or non‐cementitious. This new classification identifies the reaction byproduct as the main classifier of the material type since it is the most significant component that affects the clinical interactions. The presence of water and hydration is important; thus, it is also listed as part of the classification. Alternative vehicles that react with moisture from the environment require updated clinical protocols to ensure the hydration can proceed. The updated classification of hydraulic cement used in dentistry focuses on the clinical effect rather than on the material chemistry; thus, it is more suitable for material identification.

## 1 Introduction

Hydraulic cements require water to set and reach their optimal physical and mechanical characteristics and do not deteriorate when wet. The first use of hydraulic cements in dentistry was reported in 1993 for the use to seal root perforations (Lee et al. 1993) and as a root‐end filling material (Torabinejad et al. 1993). Mineral trioxide was patented (Torabinejad and White 1993) and the first formulation on the market was ProRoot MTA (Dentsply, Tulsa, OK, USA). ProRoot MTA is composed of 80% Portland cement and 20% bismuth oxide (Camilleri et al. 2005). The extended use of hydraulic cements to all endodontic applications (Torabinejad and Chivian 1999) necessitated the improvement of the physical and mechanical properties of the material including the ease of use and highlighted the interactions of the material with the clinical environment.

The hydration of the tricalcium and dicalcium silicate phases in Portland cement component in MTA results in the formation of calcium silicate hydrate gel and calcium hydroxide (Camilleri 2007, 2008a). The calcium hydroxide which is formed as a byproduct of hydration is responsible for the clinical effect of the hydraulic cements when used in different clinical procedures thus highlighting that the calcium hydroxide is a necessary feature of these materials for clinical success. The benefits of calcium hydroxide for vital pulp therapy have been well documented (Cox et al. 1992; Murray et al. 2002, 2022). The calcium hydroxide creates an environment which promotes the pulpal cells to form a dentine bridge. The dentine bridge only forms in close proximity to the dental pulp thus the calcium hydroxide itself cannot cause dentinogenesis (Tziafas et al. 1996). MTA exhibits better dentine bridge formation than other hydraulic cements and calcium hydroxide (Li et al. 2015; Silva et al. 2025). The calcium ion release from hydraulic cements affected the material's antimicrobial properties more than the biological characteristics (Koutroulis et al. 2019). The maintenance of a high concentration of hydroxyl ions can change bacteria's enzymatic activity and promote its inactivation. The site of action of hydroxyl ions of calcium hydroxide includes the enzymes in the cytoplasmic membrane and this medication has a large scope of action and therefore is effective on a wide range of microorganisms, regardless of their metabolic capability indicating the effectiveness of calcium hydroxide releasing materials in root canal obturation (Estrela and Holland 2003).

The calcium hydroxide released from hydraulic cements interacts with tissue fluids and dentine and has been postulated to form hydroxyapatite which has been termed bioactivity. The mechanism of formation of the phosphate from hydroxide has been described to be similar to the interaction of bioglass (Niu et al. 2014). This interaction assumes the availability of phosphates to react which only happens in vitro under a controlled environment. The interaction of hydraulic cements with the clinical environment has been shown to result in the formation of calcium carbonate on the material surface (Schembri Wismayer et al. 2016; Moinzadeh et al. 2016; Meschi et al. 2019). This was shown by characterisation of explanted material from failed cases and extracted teeth. Furthermore, the chemistry of the simulated tissue fluid led to different precipitates on material surfaces in in vitro experiments (Meschi et al. 2019) indicating that the in vitro set up could not be translated to the clinical scenario. The interaction of the hydraulic cement with the tissue fluids changes the microenvironment at the interface resulting in deterioration of the antimicrobial properties of the material due to the reduction in pH (Farrugia et al. 2017).

The diversity of materials and the clinical use led to a classification of hydraulic cements used in dentistry (Camilleri 2020). This classification describes the hydraulic cement chemistry and classifies materials based on the clinical use. Since then, more materials have been introduced for clinical use that have different chemistries. It has also been shown that most of the materials on the market are of undeclared chemistry and the quantity of each component varies largely (Cardinali and Camilleri 2023). Furthermore, the classification overlooks the hydration of these phases and the role of the byproducts which modulate the biological interactions. Due to these limitations, a new classification is being proposed. The new classification updates the classification based on material chemistry and includes recommendations for the preparation of the clinical field when using hydraulic cements.

## 2 Classification

### 2.1 Classification Based on Material Chemistry

Only materials that set by interaction with water can be classified as hydraulic cements. If the setting is through other interactions such as resin reactions, the materials are classified as resin‐based regardless of the inclusion of a hydraulic cement in the formulation.

**FIGURE 1:**
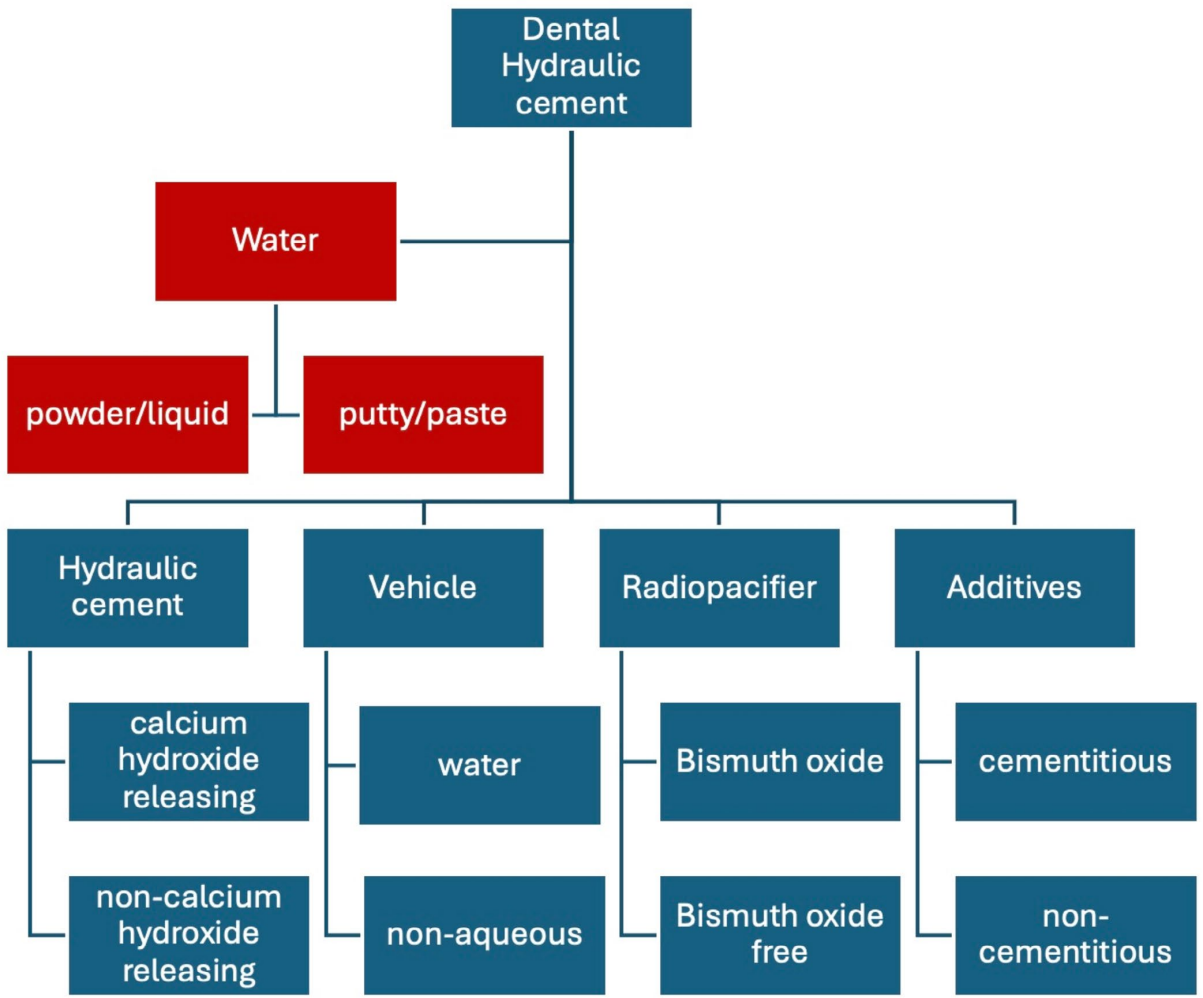
Composition of dental hydraulic cement showing the need of water to allow the material to set and the different constituents that impart the clinical properties. These constituents are subclassified as hydraulic cement, radiopacifier, additives and vehicle. The hydration byproduct is the most important identifier of the material. The hydraulic dental cements are presented as powder/liquid or putty/paste requiring environmental moisture formulations indicated in red in the figure.

All hydraulic cements used in dentistry are used with various additives. The different inclusions are shown in Figure 1. All materials require water to set. If the water is part of the formulation, it will be presented as a powder and liquid. Some materials are presented as putty or paste of variable consistency and require environmental moisture to set. These materials depend on the clinical conditions they are used in, thus are classified separately (Figure 1 red section).

Dental hydraulic cements have 4 types of components which are shown in Figure 1. These components include the main cementitious phase which allows the material to set. For materials used in endodontics, the formation of calcium hydroxide is critical as all the biological interactions are attributed to this phase. Due to this, the cements are classified as calcium hydroxide releasing or non‐calcium hydroxide releasing.

The type of vehicle is important as the materials which are presented as powder/liquid formulation and mixed with water are less susceptible to environmental changes. Materials incorporating alternative vehicles depend on environmental moisture.

All dental materials include radiopacifiers to enable detection post‐operatively. The original MTA included bismuth oxide, but other radiopacifiers are used increasingly.

The additives depend on the specific clinical use of the material. Most of the additives are non‐cementitious; thus, they are not materials that can be used on their own interacted with water. These non‐cementitious additives vary in nature and are added to enhance the physical and mechanical properties of the materials. The cementitious additives include the addition of materials that can react with water and produce a paste regardless of the hydraulic cement hydration. Such cementitious additives include alternative chemistries of hydraulic cements and calcium phosphate‐based cements.

### 2.2 Classification Based on Clinical Use

A characteristic feature of hydraulic cements is the interaction with the environment they are placed in. This has been well documented in the industrial cement literature with the effects of sea water, sewage, and other environmental effects investigated.

In the prior classification (Camilleri 2020) the hydraulic cements were also classified based on the specific use. They were grouped into intracoronal, intraradicular and extraradical materials. This classification will be retained and elaborated further by including the clinical recommendations. For dentistry, there is some research on the effect of clinical procedures such as acid etching (Kayahan et al. 2009; Camilleri 2013; Hashem et al. 2014), irrigating solutions (Lee et al. 2007; Ballal et al. 2017; Kapralos et al. 2021, 2024), and adjacent materials (Camilleri 2011a) reported. The irrigating solutions also change the characteristics of the dentine and modify the interaction of the materials to the dental hard tissues (Nagas et al. 2014). The materials are also in contact with dental hard tissues, tissue fluids and blood depending on the specific clinical use which was in fact proposed as part of the previous classification (Camilleri 2020).

Although the interactions to specific environments have been investigated, there is still limited research and guidance on how to prepare the clinical field to enhance the properties of the hydraulic cements in use. The materials that are not mixed with water and requiring environmental moisture to set are the most challenging to use as although the delivery is facilitated, they may not set. Leaving the tooth wet results in material extrusion past the root apex which is exacerbated by wide gauges and larger apical lesions (Iqbal et al. 2026). To date, the removal of smear layer seems to enable the adequate moisture levels for hydration and effective antimicrobial effects (Fernandes Zancan, di Maio, et al. 2021) with continuous chelation being the most effective way to prepare the dentine when using hydraulic cement sealers (Surana Bhandari et al. 2026). The removal of smear layer has microbiological implications (Saleh et al. 2008). No difference in bacterial leakage was noted in different thicknesses of root‐end cavities and smear layer removal when using MTA as a root‐end filling. Material (Yildirim et al. 2010). Further research is necessary to find the best strategy to match the irrigation and obturation techniques to enhance the properties of the materials used after interaction with the clinical environment (Fernandes Zancan, Hadis, et al. 2021).

The clinical recommendations for the preparation of the clinical field when using hydraulic cements in different clinical scenarios are the following:

#### 2.2.1 Intracoronal Use—Vital Pulp Therapy and Barriers in Regenerative Endodontic Therapy

The use of acid etching solutions should be kept to a minimum to avoid weakening the surface of the hydraulic cement when used to protect the pulp or as a barrier protecting the newly regenerated tissues (Camilleri 2013). Resin‐based materials are recommended for use over these layers as they do not interact with the material chemistry. Zinc oxide and glass ionomer cements should be avoided (Camilleri and Gandolfi 2010). Delaying the final restoration may be beneficial (Palma et al. 2021) and the hydraulic cement can be used as a temporary restorative material (Hashem et al. 2014). The reduction in microbial load by using sodium hypochlorite lavage over the dentine and exposed pulp prior to placement of the hydraulic cement is recommended (European Society of Endodontology 2019; Duncan et al. 2023).

#### 2.2.2 Intraradicular Use—Root Canal Obturation and Apical Closure of Immature Permanent Teeth

The removal of smear layer is recommended especially when using root canal sealers that require moisture from the environment to set. A final irrigation with water followed by canal drying ensures an optimised clinical field and avoids apical extrusion of the root canal sealer.

#### 2.2.3 Extraradicular Use—Closure of Communications With the Periodontal Ligament Space

The hydraulic cements will be in contact with blood and tissue fluids and the placement of a thick layer into a well‐prepared root‐end cavity will ensure there is no washout or material loss by macrophage action (Camilleri and Aznar Portoles 2020). The interaction of blood with the hydraulic cement results in a reduction in pH and antimicrobial activity. The resection of the apical third and adequate preparation of perforation sites ensure the reduction in microbial load in the clinical field.

## 3 Discussion

Mineral trioxide aggregates includes Portland cement which is a mixture of tricalcium and dicalcium silicates. The calcium hydroxide is only formed from the hydration reaction of the silicates (Camilleri et al. 2005; Camilleri 2007, 2008a). The presence of hydraulic calcium silicate does not automatically guarantee the formation of calcium hydroxide. In the materials using alternative vehicles to water, external factors such as interaction with blood (Oloomi et al. 2013; Schembri Wismayer et al. 2016; Song et al. 2016), other materials such as zinc oxide (Camilleri 2011b), and dry environment may inhibit the hydration reaction.

There are increasingly more materials that are composed of magnesium silicate, strontium silicate, and also calcium aluminate. Although it is assumed that these materials interact with water or environmental moisture and produce calcium hydroxide with its proven clinical benefits, this is not the case. The setting and hydration are distinct for each material chemistry, and they are in fact classified as non‐calcium hydroxide‐producing materials.

The presence of additives such as other hydraulic cements depletes the release of calcium hydroxide in the long term. Once the calcium silicate reaction occurs, the calcium hydroxide is taken up by the calcium aluminate reaction to form more calcium silicate hydrate leading to a shorter setting time (Camilleri 2008b), increased compressive strength (Camilleri 2008c) and reduced acid solubility (Camilleri 2011b) but the depleted calcium hydroxide shows a deterioration in the biological characteristics of the materials (Camilleri 2008d). Thus, mixtures of calcium silicates and calcium aluminates cannot be classified as calcium hydroxide releasing materials. There has also been some debate on the ideal amount of calcium silicate present in a material. A previous publication showed a range of 5%–50% variation in the amount of cement in the formulation (Cardinali and Camilleri 2023). Very low amounts may result in not having enough binder in the mixture with increased risk of fragmentation and loss after placement. The amount of cement in a formulation needs to be balanced with the radiopacifier percentage. Other cementitious additives include calcium phosphates in various forms which have been shown to modify the hydration and release of calcium hydroxide (Schembri‐Wismayer and Camilleri 2017). Thus, cementitious additives vary the hydration reaction.

Non‐cementitious additives vary in presentation and are included to reduce the setting time, improve the material physical characteristics such as strength, flow and film thickness depending on the specific clinical use. It is important to note that the additives may have different effects which depend on the type of cement used in the formulation. A good example of this is the use of calcium carbonate filler in Biodentine which in that system acts as a nucleating agent and together with the superplasticizer and an accelerator such as calcium chloride results in a material which uses less water thus has improved physical and mechanical properties and high calcium ion release (Camilleri et al. 2013). Biodentine uses tricalcium silicate as the main cementitious phase. Similar additives tested using Portland cement which includes aluminates resulted in reduced calcium hydroxide release due to the interactions of the calcium carbonate with the aluminates (Khalil et al. 2015). There is insufficient literature on the specific interferons of additives to hydraulic cements used in dentistry but regardless of this the current formulations have been shown to include a number of additives (Cardinali and Camilleri 2023).

Alternative vehicles include water soluble substances that easily interact with moisture. Such substances include polyethylene glycol, propylene glycol, glycerol, dimethyl sulfoxide and other water‐based gels. Different vehicles modified the hydration, physical, chemical and biological properties of tricalcium silicate cement (Son et al. 2024).

Radiopacifiers are necessary to enable detection of the material post‐operatively. MTA included bismuth oxide, which was an effective radiopacifier when used as 20% replacement of the cement. Bismuth oxide was shown to be unstable and led to tooth discolouration. This has been reported in a case series where bismuth oxide‐based materials caused tooth discolouration (Cardoso et al. 2022). The tooth discolouration is the main complication reported with regenerative endodontic therapy (Meschi et al. 2023). The change in colour is caused by a phase change and formation of the δ‐phase, which is black (Camilleri et al. 2026). Bismuth oxide interacts with collagen in tooth structure (Marciano et al. 2014), sodium hypochlorite used for irrigation (Camilleri 2014; Camilleri et al. 2020), and blood (Guimarães et al. 2015; Schembri Wismayer et al. 2016). Due to this clinical complication, the materials have been classified as bismuth containing and bismuth oxide‐free. Alternative radiopacifiers are less radiopaque than bismuth oxide, and a higher percentage is required to result in similar radiopacity as bismuth containing materials (Camilleri and Gandolfi 2010; Húngaro Duarte et al. 2009).

## 4 Conclusions

The updated classification of hydraulic cements used in dentistry identifies the clinical implications of the hydration byproducts as the main component that classifies the materials followed by the availability of water for the hydration to ensue. Materials that require environmental moisture to set should have a specified clinical protocol to enable this.

## Author Contributions

**Josette Camilleri:** conception and design; data acquisition, analysis or interpretation; intellectual content development or critical review; final version approval.

## Conflicts of Interest

The author declares no conflicts of interest.

## Data Availability

Data sharing not applicable to this article as no datasets were generated or analysed during the current study.
